# “Making our voices count”: patient action groups as a strategy to engage people with lived experience in implementation of an intervention for hypertensive disorders of pregnancy

**DOI:** 10.3389/frhs.2025.1602103

**Published:** 2025-10-23

**Authors:** Jasmine Hodges, Hiba Fatima, Kelly McHugh, Jennifer Medearis Costello, Kamara Barnett, Narges Farahi, Jessica Greene-Jones, Darci Jones, Alasia Ledford, Leila D. Lewis, Jenniqua Lopez, Shelby Smith-Janey, Mary Quezada, M. Kathryn Menard, Alexandra F. Lightfoot

**Affiliations:** ^1^ Center for Women’s Health Research, University of North Carolina at Chapel Hill, Chapel Hill, NC, United States; ^2^ Department of Maternal and Child Health, Gillings School of Global Public Health, University of North Carolina at Chapel Hill, Chapel Hill, NC, United States; ^3^ Equity for Moms and Babies Realized Across Chatham, Chatham, NC, United States; ^4^ ACHIEVE Patient Action Group, University of North Carolina at Chapel Hill, Chapel Hill, NC, United States; ^5^ Department of Family Medicine, School of Medicine, University of North Carolina at Chapel Hill, Chapel Hill, NC, United States; ^6^ Department of Obstetrics and Gynecology, School of Medicine, University of North Carolina at Chapel Hill, Chapel Hill, NC, United States; ^7^ Department of Health Behavior, Gillings School of Global Public Health, University of North Carolina at Chapel Hill, Chapel Hill, NC, United States

**Keywords:** maternal health, hypertensive disorders of pregnancy, community engaged intervention, workgroup, advisory board, engagement, implementation science, adaptation

## Abstract

Community-based clinics are a crucial site for the detection and response to Hypertensive Disorders of Pregnancy (HDP), yet many sites lack a standard protocol to address hypertensive emergencies. Moreover, interventions to address this disparity lack a patient centered lens that inform implementation in a community setting. To address these gaps, the Advancing Community and Clinical Care for Childbirth-related Hypertension: Implementation, Engagement and Valuing Equity (ACHIEVE) study is engaging patients with lived experience of HDP. The study integrates community engagement and implementation science to inform adaptation of a hypertension bundle in outpatient clinics. We have created two Patient Action Groups (PAGs) involving members from populations impacted by HDP to guide our community-academic-clinic-coalition team's iterative processes for bundle implementation and resource development. One PAG is comprised of Black and the other Latina/e birthing people, all of whom have lived experience with HDP. Both PAGs meet monthly, led by a racially/ethnically, culturally, or linguistically congruent facilitator. Agendas are co-created, and activities structured to build relationships, trust, and capacity. PAGs review ACHIEVE strategies and materials to spur recommendations and improve respectful care. To facilitate reciprocal exchange, PAG facilitators meet with the ACHIEVE community engagement team and share updates at monthly partner coalition meetings. We recommend PAGs as a power-shifting strategy to ensure those most affected by inequitable perinatal outcomes can engage in research in a way they feel safe, respected and heard. Researchers can also increase trustworthiness by listening and translating patient-generated ideas into strategies that improve clinical care.

## 1 Introduction

There is an urgent and ongoing maternal mortality issue in the United States (1). Hypertensive disorders of pregnancy (HDPs) account for much of the burden of maternal morbidity and mortality and the prevalence of HDPs is increasing over time (2, 3). A national study of maternal deaths from 2017 to 2019 found that 24 percent of women who passed had some form of HDP (2). Further, of all women that experienced any HDP, the prevalence for Black women was 21 percent, 16 percent for American Indian/Alaska Native women, 15 percent for White women, and 13 percent for Hispanic women. These trends show that the burden of disease is not equally dispersed. Similar trends are reflected in North Carolina (NC), the location of the community case study reported here, where Black women experienced the highest prevalence of maternal hypertension, 17 percent, whereas the prevalence for White and Hispanic women is 14 percent and 9 percent, respectively (4). In addition to HDP prevalence, studies have found disparities in treatment and care of HDPs, with Black women more likely to experience delays in detection and treatment of HDPs (5). Moreover, 33 percent of Black or Hispanic mothers report disrespectful care during the perinatal period (6).

Timely detection and treatment of HDPs is crucial in preventing adverse outcomes (7) as is ensuring respectful care for all pregnant people, particularly those most affected by maternal morbidity and mortality disparities (7, 8). The Alliance for Innovation on Maternal Health (AIM) developed the Severe Hypertension During Pregnancy and Postpartum Period Safety Bundle (HTN Bundle) in 2015, which initially included four R's, or procedures to improve hypertensive care during pregnancy: readiness, recognition, response, and reporting. In 2022 AIM added a 5th R for respectful care; see Table 1 for detailed description of the five R's (9). The HTN Bundle is focused on addressing inequitable outcomes by improving recognition and response to hypertension (HTN) during pregnancy by providing guidelines and protocols that can be implemented in hospital settings (7). Yet many individuals with HDPs present in outpatient settings (9, 10), making community-based obstetric and primary care clinics a crucial site for enhanced detection and response to HDP and provision of respectful care during pregnancy and postpartum. Moreover, interventions to address this disparity lack a patient centered lens that inform implementation in a community setting.

**Table 1:** The 5 Rs of the AIM severe hypertension during pregnancy and postpartum period safety bundle and corresponding PAG contributions (9).

Bundle element	Description	PAGs’ contributions
Readiness	Establish protocols and training across clinic settings to ensure teams are prepared to identify and manage severe hypertension efficiently.	Training Curriculum, Provider Conversation Guide
Recognition	Ensure prompt identification of severe range blood pressures and warning signs of pre-eclampsia through screening and accurate measurement of blood pressure.	Training Curriculum
Response	Utilize standard protocol and assessment to rapidly treat and escalate instances of severe hypertension.	Training Curriculum, Provider Conversation Guide, Respectful Care Measurement Tool
Reporting	Conduct multidisciplinary reviews and post-event (in case of escalation with severe hypertension) debriefs to improve response and performance.	Training Curriculum
Respectful care	Provide patient-centered care, engaging in open and transparent communication to ensure shared decision making and patient inclusion.	Training Curriculum, Provider and Patient Conversation Guides, Website, Respectful Care Measurement Tool

Our study, Advancing Community and Clinical care for Childbirth-related Hypertension: Implementation, Engagement and Valuing Equity (ACHIEVE), takes a novel approach to addressing these gaps. We are integrating community engagement and implementation science to inform adaptation of the AIM Bundle for the outpatient setting (completed in Phase I) and are now testing the adapted HTN Bundle's implementation, effectiveness and sustainment in Phase II (11). The goal of integrating community engagement and implementation is to reach populations that are most affected by HDPs in our region (i.e., Black, rural, and low-income birthing parents) and engage them in translating their experiences into meaningful strategies that impact our partner clinics and community (11). In this Community Case Study, we describe our initial community-engaged approach, and the changes made over time to increase involvement of individuals with lived experience of HDP and other relevant perinatal experiences through the creation of Patient Action Groups (PAGs). This article documents the principles and practices behind the establishment and maintenance of ACHIEVE's PAGs. It also highlights the impact of intentionally involving people with lived experience (PWLE) in co-creating processes and products (including this paper), and guiding the outpatient HTN Bundle procedures (i.e., 5 Rs) and implementation strategies. We will also discuss challenges encountered and highlight PAGs as a power-shifting strategy in community-academic research.

## 2 Context

The protocol of the ACHIEVE study is informed by community-based participatory research (CBPR) principles with the goal of centering community perspectives; recognizing and valuing the strengths and expertise of all partners; building authentic and equitable partnership; and promoting collaboration, co-learning and shared contributions to the creation of process and products (11, 12). One way we operationalized CBPR principles early in the project was to establish a Coalition, a community-oriented implementation strategy derived from the Expert Recommendations for Implementing Change (ERIC) compendium^1^ (13). Our coalition includes representation from the three “layers” of community central to our work (PWLE, clinic providers and staff, and representatives from community-based organizations addressing birth equity) of collaboration to inform all phases of the study (11). In addition to establishing the Coalition, we conducted formative work to understand facilitators and barriers to implementing the HTN Bundle in the outpatient setting from the perspective of clinic providers, staff and patients (14).

Drawing on the findings from the formative work and ongoing consultation with the Coalition, we made adaptations to the HTN Bundle and tested three clinic-oriented implementation strategies [Training, Coaching (i.e., Facilitation), and Simulation (13)] identified by clinic champions as appropriate for community health centers (11). These community-engaged processes in Phase I of ACHIEVE spurred focus in Phase II on additional ways to improve care of pregnant people via enhanced focus on respectful care, patient engagement and education (11, 14). This prompted the addition of two community-oriented implementation strategies [i.e., Build a Workgroup and Deliver Educational Materials (13)]. While our Phase I Coalition had representation from all three layers of community, PWLE were underrepresented; we only had one or two PWLE members, compared to having five to six representatives from each of the other layers. Moreover, in one-on-one check-ins with members, the few PWLE on the Coalition expressed their passion to be involved, yet they were uncertain about how to contribute given their low representation and the dominant voices of institutional partners. Combined with the findings from our formative work and insights shared by our partners with lived experience, our recognition of this challenge necessitated creative reimagining of how best to ensure PWLE perspectives were at the center of our work.

### 2.1 Launching ACHIEVE patient action groups

The solution proposed was to utilize the “Build a workgroup” implementation strategy (13). This complements our strategy to build a coalition because it increases the diversity of perspectives and individuals contributing, reviewing, and disseminating the work. ACHIEVE's version of a workgroup was to establish a safe, supportive, community-driven space to share birth experiences and provide crucial guidance to the research.

The concepts of our PAGs are similar to that of community advisory boards (CABs) and patient advisory boards (PABs), often used in CBPR-aligned health research. Like our PAGs, these structures are intended to ensure that community members' input and feedback drive various stages of the research process, such as adapting interventions, using appropriate data collection instruments, identifying relevant outcome measures, recruiting other community perspectives, attending trainings, and disseminating findings (15–18). Brockman et al. 2017 interviewed 17 researchers who partnered with CABs in their work, and found that the CABs were particularly influential in informing and improving study design and implementation (15). This has aligned with our experience with the PAGs. In our study specifically, PAG members lended their perspectives to educational materials, academic products, definitions of respectful maternity care for hypertension.

However, as the name Patient Action Group implies, our PAGs are action-oriented, rather than advisory. PAG members co-create tools alongside us. We intentionally chose to develop a PAG to steer our work away from pitfalls of community-engaged work that only engage collaborators in an advisory role. In such cases, feedback is, at times, solicited on an ad-hoc basis and there is little accountability to acting on solicited perspectives; researchers can accept or reject collaborator feedback (18). By contrast, our PAGs aim to meaningfully involve members in co-creating processes and products, that will help successfully implement the HTN Bundle by bridging community perspectives and outpatient settings. Along the way, we create systems to help ensure accountability to this involvement.

Our first PAG was launched a year into ACHIEVE's Phase II with direction from a newly hired Community Engagement (CE) Manager in collaboration with a longtime Coalition member with lived experience. Initially, the PAG was created for Black-identifying PWLE to engage birthing people from the population most impacted by morbidity and mortality associated with HDP. The Black-identifying (BI) PAG was launched in December 2023.

Shortly into its establishment, the Coalition member with lived experience (“BI-PAG co-lead”), recognizing that language is often a barrier to care in our region and can lead to increased health disparities (19), made the case for launching a second PAG with Spanish-speaking members. Instead of creating a new entity, ACHIEVE reached out to an existing advisory group of Latina(e) birthing people at a regional women's hospital health education center. This group was initially developed in 2018 and was created as a safe space to talk in Spanish about childbirth experiences and support improvements in the regional hospital. When approached by the CE team, the facilitator of the Spanish-speaking group (an existing coalition member) welcomed the idea of linking efforts. Work with the Latine-identifying (LI) PAG commenced in January of 2024. Partnering with this group has helped ensure the perspectives of Latine PWLE are integrated in ACHIEVE's work.

PAG leads worked with the CE Manager to develop a basic structure for convening. PAGs meet most months for up to an hour and a half via a virtual platform (i.e., Zoom). During the few months they do not meet (over the winter holidays and mid-summer), the CE Manager communicates with PAG members by email to maintain connection. Each PAG member receives compensation for the time they contribute at a rate comparable to other community-engaged projects at our research university ($65/hour). Groups are led by facilitators who are racially and/or linguistically congruent with members. The current composition of the PAGs include ten BI-PAG members from four counties in North Carolina and ten members of the LI-PAG from five counties. Both PAGs include members across rural and urban counties.

## 3 Key programmatic elements

Four commitments guide our work with our PAGs. We are committed to: respecting and valuing the expertise of lived experience, building trust among partners, embracing co-creation, and shifting power from the research team towards the community. Co-creation is defined as the “collaborative generation of knowledge by academics working alongside stakeholders from other sectors” (20). Greenhalgh and colleagues outline three elements that uphold effective co-creation. These are 1) understanding the system within which you are operating, 2) viewing the research process as a creative endeavor while also centering the human experience, and 3) approaching relationship-building and power sharing as an ongoing process (20). Operating with these co-creation elements helps ensure that our collaboration with the PAGs is intentional, respectful, trust-based and facilitates greater power-sharing with our community. With respect to co-creation and trust as guiding principles, our key programmatic components include recruitment, agenda and facilitation, co-design and adaptations, and feedback and reinforcing trust by acting on what is discussed.

### 3.1 Recruitment

Our primary recruitment strategy to build our first PAG (BI) was inspired by Greenhalgh's third element of co-creation- continuous relationship-building to share power (20). To do so, we started by identifying existing partnerships through our Coalition, as many coalition members are involved in trusted networks that collaborate with PWLE. We asked coalition members to recommend individuals that might be interested in joining the PAG. This allowed us to start connecting with people that had similar interests in perinatal work. We followed up with coalition members via email with a flyer and link to an interest form that we created so they could easily share the message with their networks. We also shared flyers in community newsletters and posted them in local partner clinics. We reached out to participants in community events we hosted in different counties where ACHIEVE is working called Story Circles, where we invite birthing people and birth professionals to a local community space to discuss their experiences related to HDP and respectful perinatal care. Following such an event, we make sure to share additional opportunities for participants to continue to engage with ACHIEVE. This strategy resulted in recruiting the ten members we now have in our BI-PAG.

To build connection and shared purpose, and, in turn, increase the chances of attendance for individuals that complete the interest form, the CE Manager hosts one-on-one relational meetings with potential participants. These meetings help to further communicate the purpose of the group and the project; it also helps the manager introduce herself since she collaborates heavily with the groups. These meetings demonstrate our relational approach and set the foundation for power-sharing in the future. Once members have started attending meetings, they are asked if they have any recommendations of individuals that may be interested in joining the group. We keep the interest form open on a rolling basis and remind our networks of this collaboration opportunity quarterly.

Throughout the early stages of our work, clinical and community partners highlighted the need for Spanish-speaking patient engagement. As previously mentioned, we employed a different approach to gather this group, collaborating with an existing work group of Latina(e) birthing people through a local health education center, which had initially met every other month and built trust during COVID-19. This group also recruits members on a rolling basis.

### 3.2 Agenda and facilitation

**Figure 1:**
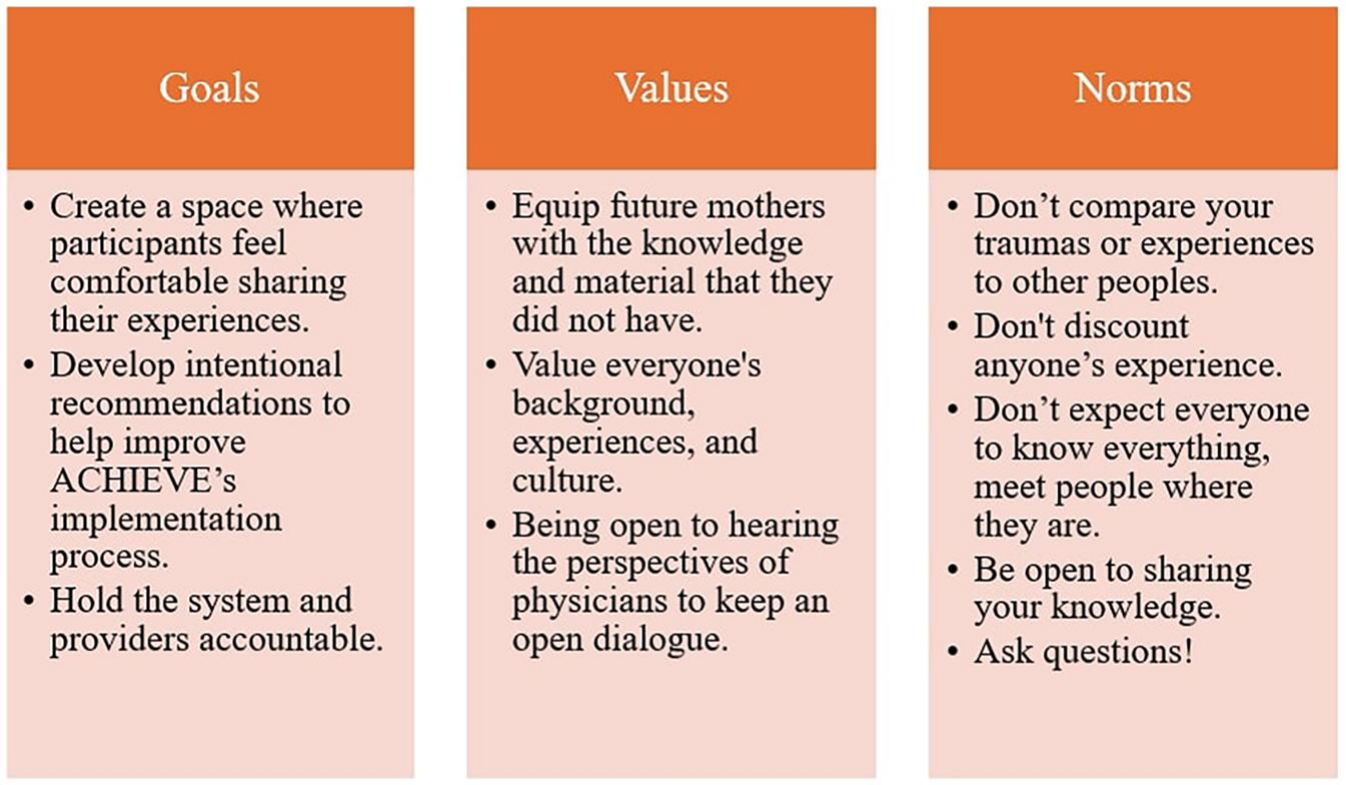
BI-PAG identified goals, values, and norms.

When the PAGs first began to meet, facilitation was primarily focused on understanding the work of ACHIEVE and the purpose PAG workgroups could serve. The groups were asked to co-design a team charter, which we refer to internally as our guiding document. The goal of this document was for members to define the objectives, goals, and values of the work group. At the beginning of each meeting, facilitators include a PowerPoint slide that shows the goals and values as a refresher; this document is also revisited with the PAGs once a year to accommodate for any changes the group wants to make (see Figure 1 for the complete list of BI-PAG goals, values, and norms). This process allows us to intentionally build relationship within the PAGs while also making space for collective growth and shift in priorities and values (20).

From our discussions in early meetings, we learned that the groups value a routine meeting flow. This has been important to the reception from the PAGs; how the facilitators organize, and lead meetings can be determinants of engagement and growth. Each meeting follows this general format: announcements, icebreakers, presentation of content, and discussion. This format helps us to optimize engagement with members, as it allows us to have multiple points of interaction throughout the meeting. It's important to note that these meetings are patient-centered and patient-led, meaning that if there is certain content or questions that we do not reach due to discussion, we do not rush the group. The pace and flow of the conversation is determined by members. Taking this into account, it is inevitable that we sometimes run out of time or cannot allocate sufficient time for all agenda items, whether this is due to prolonged discussion or technical difficulties (e.g., video chat crashing). To address this, we make sure that a missed item is moved to the next meeting agenda; if we run out of time on an item, we always follow up asynchronously through email to allow members to share remaining discussion and ideas.

### 3.3 Co-design and adaptations

When it comes to determining the specific content of the agendas (i.e., ACHIEVE products and processes for consideration), we have an internal submission process that allows study staff to request space on the agenda to present their content to the PAGs for community review. Once staff submit their request, the CE Manager schedules and buildsout an agenda, communicating expectations to the presenters around engagement and discussion. Since the PAGs' inception, members have co-created and reviewed multiple products ranging from clinic training slides to public facing website content. This collaboration not only positively impacts our implementation strategies but also informs how HTN Bundle procedures (5 Rs) are tailored with community insight. Below are descriptions of various products that have been developed in collaboration with PAG members. Please see Table 1 for how each of these products corresponds with the 5 Rs of the adapted HTN Bundle.

In one of the first meetings, ACHIEVE's Nurse Coordinators, responsible for carrying out clinic-oriented implementation strategies [i.e., Training, Coaching (i.e., Facilitation), Simulation] (13)) for HTN Bundle implementation, presented their clinic training curriculum around taking blood pressure and respectful care for medical assistants and nurses at our partner clinics. At the time, the slides had not yet been reviewed by PWLE; in reviewing them, PAG members gave insightful feedback on what respectful care behavior looks like for them in this setting. Behaviors they recommended the coordinators integrate in training included: making sure the provider introduced themselves when they enter the room, asking if patients had any questions during the appointment, and routinely having an interpreter available at the patient's request. Prior to receiving this input, Nurse Coordinators had only listed the definition of respectful care, as provided by the World Health Organization, in the training. Adding specific behaviors identified by PAG members as crucial elements of respectful care helped make the training more patient-centered, robust and tangible to clinicians, allowing clinicians to understand what respectful care looks like in practice specific to their patient community.

**Figure 2:**
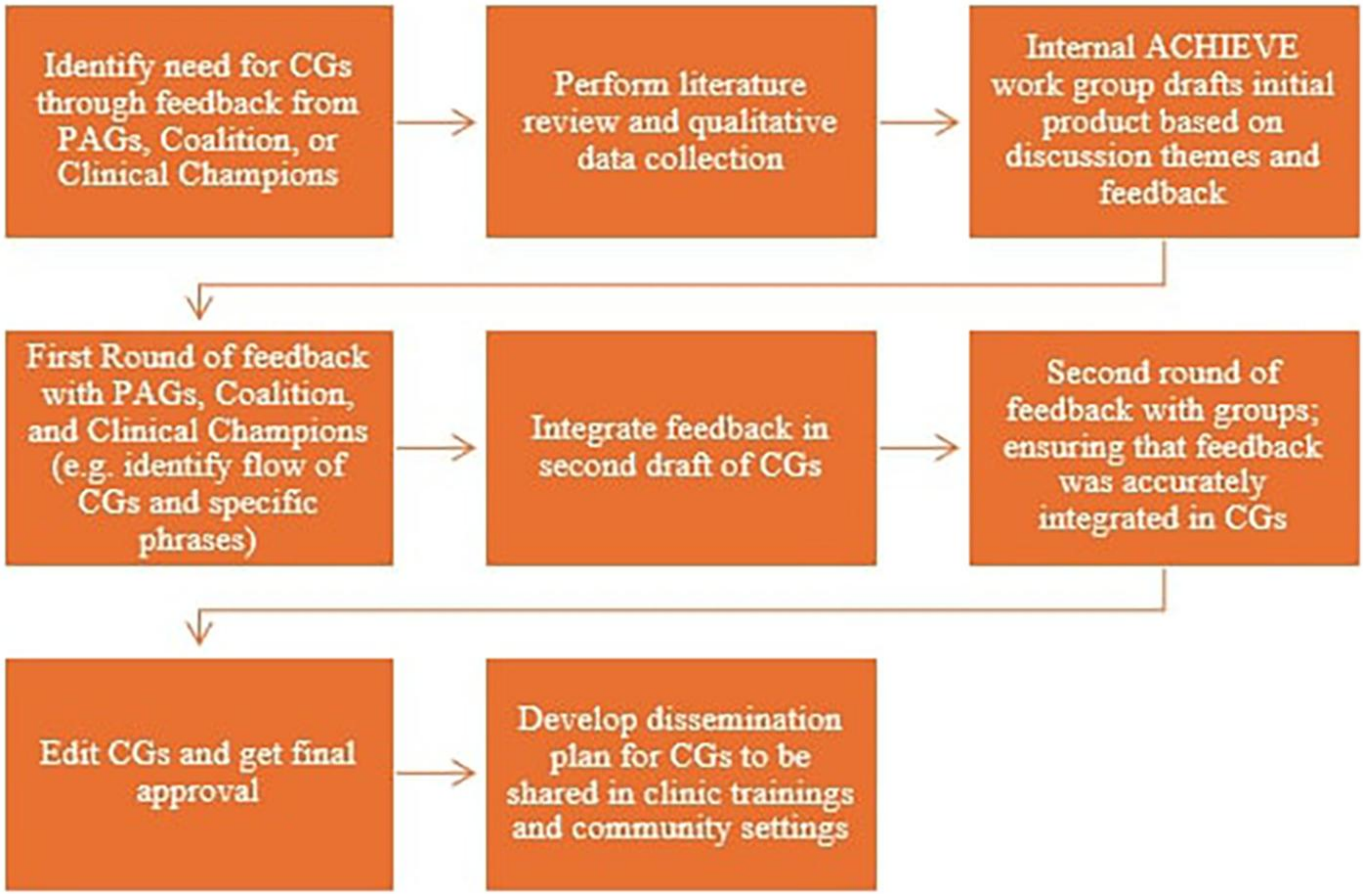
Journey of an ACHIEVE product: conservation guides (CG).

The second community-oriented implementation strategy added in Phase II, as noted above, was “Deliver Educational Materials” (13). Our PAGs have played a crucial role in developing these materials. As one example, our team worked on a series of conversation guides with the PAGs. The guides are a tool to enhance respectful, bi-directional communication related to HDP and care escalation (i.e., if a patient needs to go to the hospital). Partners recommended the creation of three complementary guides for providers, patients, and front desk clinic staff (who set the tone for patients as they enter or call the clinic). ACHIEVE staff used principles of community-based participatory research and methods of human-centered design thinking, an approach that places users at the center of the design process, to develop the guides (21). They hosted multiple meetings with the PAGs to co-create the documents in parallel with sessions that included the broader coalition and providers with experience in maternity care. PAG members contributed to the initial conceptual design, brainstormed the content (e.g., phrases, scenarios) of the guides, and iteratively refined the precise phrasing to align with what they would like to hear from their own physicians. Further, some PAG members attended in-person simulations that were used to further refine the content. See Figure 2 for an illustration of the journey of an ACHIEVE product. Overall, PAG members focused on ensuring that providers create space for patient questions, and that patients feel equipped with language to self-advocate in an emergency situation.

The PAGs have also contributed to the communication efforts of ACHIEVE. Over the last year, ACHIEVE's communication team has been working to develop a public-facing website that houses project details, clinic resources, education materials, and general information. To ensure the community-facing content was relevant to community needs, the ACHIEVE Communication Specialist attended a PAG meeting to discuss readability of the homepage, additional resources members would like added, and educational facts that were missing. As a more specific example, the PAGs mentioned that there was a high level of academic jargon and recommended to make it more reader friendly by “using language a third grader can understand.” Additionally, members suggested we add direct and easily accessible education around the signs and symptoms of HDP. Initially, we had a link to a document that outlined this information, but PAG members emphasized that too many clicks could be a barrier and that it is not a guarantee someone will click the link. In response to this feedback, our website now provides a detailed overview of the information (including critical information from the document) that is immediately accessible in addition to the link to the physical resource. PAG suggestions have helped us shape the educational parts of our website to be more accessible and direct with the resources we are providing to the community.

PAG members also collaborate in disseminating the work of ACHIEVE. As mentioned above, this manuscript was developed collaboratively with ACHIEVE staff and PAG members. Prior to drafting this case study, we submitted an abstract to the 2024 American Public Health Association (APHA) Conference which was accepted. The title of the presentation was *Building Trust is Not Linear: Creation of ACHIEVE's Black and Latina/e Patient Action Groups* and it was co-presented by the CE Manager and BI-PAG Lead. In preparing for the APHA presentation, they facilitated a feedback session to garner input, guidance and approval on content and flow. PAG members provided crucial feedback that strengthened the presentation. For example, during the meeting, members suggested we sharpen how we explain the purpose and impact of the PAGs, specifically recommending we talk about how the PAG derived from the coalition and use direct quotes from PAG members to emphasize impact. PAG members made these changes because they wanted our audience to know the feasibility of creating a PAG and the benefits to community members. To integrate this feedback in the presentation, PAG members were specifically asked two questions: “If an organization is interested in starting a PAG, what would you want them to know?” and “from your personal experience, what is the value of participating in a PAG?” PAG members' experiences and insights were incorporated into the final presentation, ensuring that their perspectives were shared with a wide audience.

Most recently, PAG members have played a crucial role in refining a respectful care reflection tool, piloted in Phase I (11). The pilot tool was created by the research team in response to a gap in tools to measure respectful care (22) Guided by the Research Prioritization by Affected Communities (RPAC), a method used to directly engage communities in identifying and prioritizing healthcare research questions (23). ACHIEVE has worked closely with our PAGs to develop a community informed respectful care measurement tool. This has involved two work sessions where PAG members defined and prioritized observable behaviors related to respectful care. The tool, when completed, will be used by clinicians to score clinical simulations, where a standardized patient interacts with a provider on HDP care, and assess them on respectful care behaviors demonstrated. PAG members will also score a subset of the video recorded clinical simulations. A description of the specific RPAC methods used to develop this tool and identified behaviors will be described in depth in a forthcoming paper.

The PAGs provide invaluable perspectives and collaboration. Given this, one of our concerns has been their feedback potentially being “lost in translation” or diluted when communicated to the broader ACHIEVE team and clinics. To limit this “dilution,” we try to incorporate strategies that allow for iterative and collaborative review. This includes monthly report outs (see Section 3.4) and having additional meetings for members to review changes we make to products based on their feedback to ensure we heard them correctly. We also do not silo PAG members to only be a part of the PAG, instead inviting them to join our coalition and standalone community events (e.g., Story Circles, Ripple Effect Mapping), environments where we are sharing work and feedback from the PAGs. This enables members to see how their feedback is shared and correct as necessary. We hope to communicate to members and the broader ACHIEVE team that the PAG's perspectives are necessary every step of the way.

All together the above demonstrates our commitment to Greenhalgh's second principle of co-creation: allowing space for creativity and centering human perspectives. The PAGs shared their feedback and review on all these products, thus infusing our work with not only the experiences and perspectives of PWLE but also their creative and technical feedback on how to improve our work. (Greenhalgh) Moreover, these varied activities of the PAG demonstrate the action-oriented nature of their role beyond an advisory context.

### 3.4 Feedback and reinforcing trust

The experience PAG members have with ACHIEVE is just as important as the ideas they contribute to the work. We have two primary methods to solicit feedback about their engagement. The first is informally, during PAG meetings. In our work, our goal has been to create a space where individuals feel comfortable sharing their feedback informally, having multiple check-ins where we ask if there are any changes we should make to our approach. However, we recognize that not everyone feels comfortable offering this level of feedback in a group setting, so we also have an anonymous survey. We send this survey to all of the PAG members once a year and ask about what they have enjoyed and what improvements they would suggest. When asked what they enjoyed about the PAG, a LI-PAG and BI-PAG member, respectively, offered these reflections: (See Table 2 for additional insights from PAG members):

**Table 2:** Feedback from patient action groups (July 2024).

Survey question	Black-identifying PAG	Latina-identifying PAG *(Translated)*
What do you enjoy about the PAG?	I’ve enjoyed the open dialogue with the other moms in the group.	Being able to help other women to feel complete trust/confidence with their doctor
	I enjoy providing feedback that could help a future doctors provide better service for moms to be.	This is a great way to improve services and for us, the patients, to better understand healthcare
	I feel like the recommendations and advice I am giving is being considered and not just a waste of my time	How much we have learned, from the experiences of the other women, and the community built in the meetings​
	Validation in thoughts and opinions about how the patient experience can/should be different,	That we, as the Latina community, are heard, and the opportunities that it has for all the people, allowing us to be able to share.
	I love the empathy from the individuals in the group.	I like that they allow us to express ourselves freely, that they are empathetic in listening to our opinions.​
What Improvements would you Suggest?	It would be nice to have more women participate but I understand that it is not at the control of the achieve team.	The only thing would be if the group could meet at a time when all of us were available, to be able to participate more and as a result have more ideas
	If I had to pick something it would be the flow of the meeting but that would me just nit picking!	That there were more of us.
	While this is not a job to be formally compensated perhaps the stipend/gift could have been a little more substantial to reflect the value of the input.	Perhaps we could meet more often so that the projects could progress/move forward more each day
	I would suggest starting every meeting off with an ice break to create more room for the individuals to get more comfortable with each other.	I would like if we broadened the topics of conversation, not just within the maternal health area…

“Me gusta que nos permiten expresarnos libremente, que son empáticos en escuchar nuestras opiniones y buscar adaptarlas a sus proyectos. Valorar nuestra opinión y hacer valer nuestra voz es muy poderoso y valioso.

[translation: I like that they allow us to express ourselves freely, that they are empathetic in listening to our opinions, and they work to adapt them into their projects. Valuing our opinion and making our voices count is very powerful and valuable]”

“I have enjoyed providing feedback that could help a future doctors provide better service for moms to be. I feel like the recommendations and advice I am giving is actually being taken into account and not just a waste of my time.”

As evidenced in the quotes above, common reflections of what participants enjoy include, feeling heard, building community with other PWLE, validation of opinions and experiences, and being able to help future mothers' healthcare experience. When asked about what improvements can be made to their experience with the PAGs, members suggested increasing the number of participants, extending the meeting time and frequency, increasing compensation, and following up on materials they had given feedback on.

A part of building and reinforcing trust is acting on what we say and what we are being told by PAG members. While some of the improvements suggested by the PAGs are not immediately actionable (i.e., increased compensation and varying member attendance), we incorporated those changes we could make right away into our operations. For example, we have instituted report outs at each meeting to share the status of products we have co-created with the PAG, and how they have been or will be used. Report outs demonstrate that we are integrating and acting on PAG members' contributions. These reports provide an additional layer in our iterative process of follow-up and hold our team accountable to the PAGs as a means of sharing power (20).

Another way we have responded to PAG feedback is through having additional recruitment pushes to expand membership. Though we keep our interest form open on a rolling basis, we have started to actively recruit (i.e., re-posting flyers, re-sharing the opportunity to partners, conducting relational meetings when interest is shown, expanding geographical recruitment as the study moves into different counties) at multiple points during the year. In the future, to respond to our PAGs' request for more time together, we plan to extend the meetings by 30 min for members that would like to stay longer. Finally, our budget is renegotiated once a year. We will also request an increase in compensation for PAG members next time we revise the budget.

## 4 Discussion

Ample studies highlight the importance of meaningful community engagement to address vexing public health problems like HDP. Translating an evidence-based intervention such as the outpatient HTN bundle necessitates intentional co-creation with PWLE to improve the quality, efficacy and patient-centeredness of products and interventions. This case study adds to the literature by summarizing PAGs' contributions to the development of multiple products and processes as well as actions taken in the establishment and maintenance of two PAGs. This section provides gives insight into the impact of PAG engagement, as well as our lessons learned for those seeking to improve research outcomes through intensive and mutually beneficial community engagement.

### 4.1 PAGs as a power-shifting strategy

Based on our experiences in ACHIEVE, PAGs are a promising power-shifting strategy to ensure those most affected by inequitable outcomes become respected members of the research team. To further demonstrate how our PAG-related activities *actually* shifted power, in this section, we compare them with the seven core competencies for power shifting in CBPR as identified by researchers Ozano et al. (2024) (24). Though our work with ACHIEVE is not fully structured as CBPR, we find these competencies relevant in examining how our activities transferred power among researchers and PAG members. See Table 3 for a full list of these competencies.

**Table 3:** Seven core competencies for power sharing as developed by Ozano et al. 2024.

Competencies for power sharing
Capacity to interpret and respond to individual and relational identity, connection, uniqueness and inequities
Ability to work closely with communities in the most suitable, inclusive and synergistic way
Aptitude for generating safe and inclusive spaces for multidirectional knowledge and skills exchange that goes beyond the research focus
Expertise in democratic leadership and/or facilitation to balance competing priorities and ensure shared decision-making
Capacity to analyse readiness for action, successes and areas for improvements throughout the research process
Ability to instigate sustainable change processes within the political dimensions of systems, policies and practices using advocacy, lobbying or activism approaches
Skills to interpret and disseminate findings and outputs that are understandable, respectful and promote community ownership

Additionally, for engagement to be power-shifting, it must include meaningful investment and dedication from the research team. On ACHIEVE, this has looked like investing energy, time, and being responsive to PWLE, sometimes changing the direction of a product or the timeline. One example of this was the initial creation of the PAGs when individual PWLE voiced the value of a separate space fostered a relational experience where safety and respect were ensured. While our original plan included a cross-sector coalition of people working in maternal health (e.g., doulas, breastfeeding experts, midwives, clinic leadership and physicians) alongside individuals with lived experience, we were able to shift in response to crucial feedback, and add one, and then, a second PAG. The formation of these additional workgroups could be considered an example of the power shifting competency described by Ozano as “responding to relational identity, connection, uniqueness, and inequities” (24).

Indeed, one of the most satisfying results of our collaboration has been the impact of PAG members on more domains of the project than what was originally planned. In addition to attending PAG meetings, several members participate in the Coalition or serve as standardized patients in HDP clinic simulations (an ACHIEVE implementation strategy). Their deepened familiarity with the work has strengthened their guidance and expanded their engagement. These practices are an example of how our processes shifted power by “generating safe and inclusive spaces for multidirectional knowledge and skills exchange that goes beyond the research focus” (24).

In doing so, we have found it necessary to ensure balance across the different layers of community (i.e., the PAGs, community coalition, and clinical partners). With PWLE working in predominantly one space and other community partners (e.g., clinical staff) in another; the research team is responsible for communicating findings from one group to another. In that process, we were concerned that community voice could be diluted or “filtered” before reaching decisionmakers. While we are still contending with this possibility, we have identified key processes to ensure PAG members “check our work.” As mentioned in Section 3, the iterative reporting out to the PAGs of what information we are relaying to other partners as well as how PAG contributions impact adaptations to ACHIEVE implementation strategies is an example of creative ways we strive for reciprocity and co-learning across the layers of community.

Beyond impacting the learning and products on ACHIEVE (the “what”), the expertise of PWLE has also impacted the “how” of ACHIEVE's work. ACHIEVE research team members outside the CE team came to see the value of reciprocal engagement and began to ask more for perspectives of PWLE on different components of the work. The approaches we use within the Coalition and PAGs to build trust and shared decision-making on the project have been taken up by ACHIEVE staff and incorporated as practices across the broader team. These approaches are also described by Ozano et al. As a power shifting competency, specifically demonstrating “expertise in democratic leadership and/or facilitation to balance competing priorities and ensure shared decision-making.” (24). Examples include altering meeting agendas to make space for participants to get to know each other, investigating our positionality in the context of our work, and expanding the size of team meetings to ensure more voices are incorporated in decisions.

We plan to use these seven core competencies to guide reflection and action on how to continue towards shifting power to our PWLE partners. For example, we hope in our next steps to expand our advocacy to systems change, in line with the competency of “Ability to instigate sustainable change processes within the political dimensions of systems, policies and practices using advocacy, lobbying or activism approaches” (24) and to co-create a dissemination plan for our findings with PAG and coalition members as suggested by the “Skills to interpret and disseminate findings and outputs that are understandable, respectful and promote community ownership” competency (24).

### 4.2 Lessons learned

Adding PAGs was an innovative investment within our ACHIEVE project that demonstrates our research team's support of and commitment to the community. Our funder's emphasis on community engagement enabled us to build in necessary financial investments and adjust them as needed. These included: a full-time CE Manager focused on supporting the PAGs and the Coalition; two part-time Community Engagement co-investigators; two part-time graduate research assistants; compensation for the time of PAG members and other community-based leaders; and childcare and meals to support in-person sessions.

We recognize that not every study will have the budget to support community engagement at this level. Studies with limited funding can demonstrate meaningful, reciprocal engagement by publicly recognizing the expertise and skill partners bring to the work, or offering opportunities for partners to advance professionally or personally (25). For example, we offer our PAG and coalition members professional development opportunities to build skills (i.e., research dissemination), complement their existing expertise (i.e., facilitating community-oriented events), and expand their networks (i.e., attendance at annual networking meetings of funders, etc.) Below are additional lessons learned that can assist future studies in maximizing and honoring community engagement in their research efforts.

As stated above, in our recruitment, we teamed up with an established patient/community group to serve as the LI-PAG because our original ACHIEVE research team did not include a Spanish speaking facilitator. Overall, this proved to be a good solution. We did, however, continue to face challenges ensuring that both PAGs have parallel opportunities for engagement. Because we are limited in the number of internal ACHIEVE staff who are bilingual, we are sometimes reliant on waiting for ACHIEVE products to be translated before the LI-PAG is able to provide input. In one such case, because of this delay, the BI-PAG gave three rounds of feedback on a product before the Spanish-speaking PAG was able to review it once. Going forward, the facilitators of the two PAGs are developing plans to ensure their PAGs have more equitable, parallel and timely opportunities to guide ACHIEVE's work.

Related to facilitation, one element that PAG members highlighted as enriching their engagement was the individual-level approach taken by the CE Manager and co-leads to bridge community and research, noting that facilitators “created a safe space and community” within the PAG. The CE Manager took additional measures to ensure the PAG members' experience was positive, including pausing the planned agenda to make space for other important issues or needs as they emerged. Meetings and events intentionally included fun and lighthearted community building to improve connection—ice breakers, shared meals, goodie bags, and activities for members' children during in person events.

During our final Phase I ACHIEVE Coalition meeting where we reflected on what we had achieved, community members emphasize that in our work with the community, we must “move at the speed of trust.” This is a value we have brought forward into Phase II and emphasized in our work to build and reinforce trust with our PAGs, though doing so has revealed inevitable tension points between traditional research processes and intentional partnership. We encountered tensions with the traditional, top-down academic approach that is embedded in research institutions (12, 26)—especially expectations for reporting, developing manuscripts, creating educational materials, and submitting budget requests—which added pressure to expedite the work with the PAGs. For example, before we could start reviewing and co-creating materials with the PAGs, we had to make sure that there was a foundation of trust and understanding of the work we were doing, which ultimately delayed certain products from being completed in the expected timeline. The opposite also occurred: bureaucratic academic systems around compensation or limited staff hours sometimes delayed our ability to follow through or honor requests made by the PAG.

In the end, slowing down to “move at the speed of trust”-not only positively altered the experience for PAG members and the research team, but also opened our eyes to unanticipated short-term outcomes. With our PAGs, two such outcomes included: 1) participants who had not previously been provided information about hypertension during pregnancy gained understanding of the condition; and 2) clinic partners were able to return to their clinics with PAG-vetted education materials to distribute before the end of the ACHIEVE project.

## 5 Conceptual or methodological constraints

Despite the many strengths described throughout this case study, we did encounter several challenges pertaining to recruitment and retention of PWLE to guide the work of the PAGs.

Central North Carolina—the region in which ACHIEVE operates—is home to multiple research universities. Residents in neighboring communities are frequently recruited to participate in research projects, sometimes even participating in multiple at any given time. Additionally, research institutions have legacies of exploiting and extracting information from neighboring communities—particularly Latina(e) and Black communities—without necessarily returning benefits to the community (26, 27). This not only adds a layer of over saturation, but also a legacy of mistrust that can create hesitation among community members to engage in research efforts. This legacy of research exploitation is also one of the reasons it is so urgent to execute a co-design strategy, shifting the power of content creation and collaboration into the hands of PWLE. The PAGs have been ACHIEVE's central strategy for doing so without the presence of academic and clinical partners.

Our recruitment strategies for the PAGs—which mainly involved going through community partners—may have disproportionately led us to engage birthing people in the region who are, at minimum, already more socially connected to support services in their county. This may have resulted in us recruiting individuals whose Socioeconomic status (SES) may not reflect/represent that of the broader population in the catchment areas of the clinics. While SES does not protect Black and Latine birthing people from experiencing discrimination and medical mistreatment (28, 29), we do also want to partner and learn from those who face additional SES-related barriers to HDP care and support. Finally, our PAG members have had varying capacities to engage, with not all members able to consistently attend meetings. We sometimes struggle to elevate the voices of those who have lower capacity. We have an opportunity as we move into further counties to intentionally recruit for varied experiences and ensure we are creating multiple pathways for participation. Nevertheless, overall, PAG members have said that they see it as a strength of the PAG that there is already a diversity of experience both within and across the two PAGs.

## 6 Conclusion

Adapting and implementing evidence-based practices into a community setting calls for intentional collaboration with PWLE to ensure perspectives of those most impacted by the health challenge inform the implementation process. Working in partnership with the PAGs, we drew on a shared dedication to improving the outcomes of birthing people with hypertension. Because our study is clinic facing to enhance and improve clinical practices, we do not know how PAGs will directly impact clinical HDP outcomes. However, we can attest to the ways that their partnership has enhanced our research processes, integrated PWLE perspectives into products, informed trainings intended to improve clinical practice, and contributed to tools that will assess patient care, emphasizing the crucial role community engagement can play in implementation science.

## Data Availability

The original contributions presented in the study are included in the article/Supplementary Material, further inquiries can be directed to the corresponding author.

## Footnotes

^1^The ERIC Compendium provides a refined compilation of 73 strategies aimed to standardized terms and definitions used to improve implementation science and clinical practice (13).
